# Global Sensory Qualities and Aesthetic Experience in Music

**DOI:** 10.3389/fnins.2017.00159

**Published:** 2017-04-05

**Authors:** Pauli Brattico, Elvira Brattico, Peter Vuust

**Affiliations:** Center for Music in the Brain, Department of Clinical Medicine, Aarhus University and The Royal Academy of Music Aarhus/AalborgAarhus, Denmark

**Keywords:** music aesthetics, neuroaesthetics, musical features, naturalistic paradigm, visual aesthetics

## Abstract

A well-known tradition in the study of visual aesthetics holds that the experience of visual beauty is grounded in global computational or statistical properties of the stimulus, for example, scale-invariant Fourier spectrum or self-similarity. Some approaches rely on neural mechanisms, such as efficient computation, processing fluency, or the responsiveness of the cells in the primary visual cortex. These proposals are united by the fact that the contributing factors are hypothesized to be global (i.e., they concern the percept as a whole), formal or non-conceptual (i.e., they concern form instead of content), computational and/or statistical, and based on relatively low-level sensory properties. Here we consider that the study of aesthetic responses to music could benefit from the same approach. Thus, along with local features such as pitch, tuning, consonance/dissonance, harmony, timbre, or beat, also global sonic properties could be viewed as contributing toward creating an aesthetic musical experience. Several such properties are discussed and their neural implementation is reviewed in the light of recent advances in neuroaesthetics.

## Introduction

When the legendary music producer Phil Spector created the trademark “Wall of Sound” aesthetics during the 1960s, the point was not about music theory or song writing, or even about instrumentation, but something abstract yet firmly anchored in the world of sense: he wanted to create a saturated, dense sound that would be aesthetically appealing even when played out from the monoaural AM radio and jukebox devices of the time. Similar conclusions can be made on the basis of observations of audio and sound engineers who likewise work with abstract sonic notions that, somewhat paradoxically, refer to concrete sensory experiences. A guitar sound, for example, can be “thin” or “full”; a drum must be “singing out,” “wide-open,” “cool,” “not muffling,” “pretty tight,” to have “a little more of a smack” (Porcello, 2004, pp. 741–744).

Provided that such qualities are aesthetically important, and well-known and much used by musicians, what are they? To first coin a heuristic term, we propose to call them *global sensory qualities*. What we mean by saying that they are global is that they concern the “whole sound” distinct from any of its individual parts, instruments, harmony structure, intervals, melody, or tuning. Moreover, many or at least most of these musical qualities seem to refer to sensory qualities. For example, when a snare drum is characterized as “pretty tight,” the notion does not seem to single out a particular affective or cognitive property, let alone a property grounded in (Western or non-Western) music theory. From the context, it is clear that what is at stake is a snare drum sound not spread too wide in terms of its sensory-related acoustic dimensions (space and reverb, frequency, timbre, sustain) in order to “sit well” in the whole mix and thus to emerge distinctive enough amongst the background of other materials. In short, the global sensory properties are both global properties, in that they concern the whole percept, but also sensory-based, since they seem to describe sensory qualities.

The premise of the present article is that global sensory qualities constitute an important yet neglected factor in a musical aesthetic experience, and could provide a fruitful avenue for research into the psychology and neurobiology of aesthetic perception. For instance, we propose that these global features are statistically extracted from the stimuli by the auditory system—or, perhaps more likely, by some subsystems (McDermott and Simoncelli, 2011; McDermott et al., 2013)—and then passed on to high-level processing, ultimately leading to the main outcomes of musical experience, namely aesthetic judgment, emotion and conscious liking, or preference (Cela-Conde et al., 2011; Brattico et al., 2013).

The idea itself is not new, especially what comes to visual aesthetics, but rarely applied to music. The notion that there are global visual sensory qualities triggering an aesthetic response has a long history, as argued for example by Bell (1914) in his theory that successful (visual) art involves a “significant form” leading to universal aesthetic experience and emotion. For Bell, the significant form, whose ultimate nature he left mysterious, consisted of “combinations and arrangements” of various visual elements such as lines, form and shapes. He wrote that “forms arranged and combined according to certain unknown and mysterious laws do move us in a particular way, and that it is the business of an artist so to combine and arrange them that they shall move us” (loc. 184).

Vision scientists have not shied away from searching for Bell's significant formula for aesthetic experience, and, recently, a number of them have tried to locate the form in global sensory properties. Jacobs et al. (2016), for example, examined aesthetic judgments of various visual textures and argued that they correlate with global computational properties, such as the presence of lower spatial frequencies, oblique orientations, higher intensity variation, higher saturation, and overall redness. By examining industrial design and visual aesthetics, Hekkert (2006) proposed four sensory qualities that can increase the aesthetic appeal of an object: (i) maximum effect for minimum means (“economic computations are favored over more complex ones”); (ii) unity in variety (“ability to see regularities and patterns in complex observations”); (iii) most advanced, yet acceptable (“the correct balance between novelty and repetition”); (iv) and optimal match (“information from different sensory modalities should converge with each other”). Renoult et al. (2016) found out that the (algorithmically modeled) sparseness of the activity of simple cells in the primary visual cortex (V1) correlates with female face attractiveness when assessed by male participants, suggesting that there might be general, non-face recognition specific neuronal properties that factor into facial aesthetic evaluation. Spehar et al. (2015) reached similar conclusions by correlating visual sensitivity with the aesthetic properties of visual random patters. Other candidates for global sensory properties that have been studied recently include processing fluency (Reber et al., 2004; Babel and McGuire, 2015; Forster et al., 2015), distribution of spectral frequency power (Menzel et al., 2015), self-similarity and fractal properties (Taylor et al., 1999, 2011; Spehar et al., 2003; Hagerhall et al., 2004; Mureika et al., 2004; Graham and Field, 2007; Redies, 2007, 2015; Forsythe et al., 2011; Mallon et al., 2014).

Could similar properties play a role in determining aesthetic responses to music, and could this hypothetical causal relation be pinpointed accurately? In the following sections, we argue that this is likely the case and propose hypotheses to be tested in future research, complementing the current focus on more local factors derived from music theory. Indeed, global features constitute but one subset of auditory features relevant to music, along with others (e.g., pitch, timbre, intervals, harmony, melody, music syntax, and individual instruments), much studied both in connection with auditory processing in general (see e.g., Koelsch, 2011), but also in connection with aesthetic perception (for reviews, see Nieminen et al., 2011; Brattico and Pearce, 2013; Brattico et al., 2013; Hodges, 2016). Perhaps global sensory properties play even a special role in musical pieces of pop/rock/metal genres, in which harmony and voice leading rules are often violated but music producers follow specific professional principles toward reaching a defined aesthetic goal (Račić, 1981; Baugh, 1993; von Appen, 2007). Today almost all music is produced, recorded, reproduced and consumed electro-acoustically, and has become a ubiquitous experience in our everyday lives. Musical pieces that resemble classical music styles, such as film soundtracks (Huckvale, 1990) or computer game music (Bridgett, 2013), are today composed and produced with computers. While historically musical aesthetics has concentrated on the classical music genre, more recently also pop/rock and jazz music has received attention by aesthetic (von Appen, 2007; Juslin et al., 2016) and neuroaesthetic scholars (Limb and Braun, 2008; Janata, 2009; Berns et al., 2010; Brattico et al., 2011, 2015; Johnson et al., 2011; Montag et al., 2011; Pereira et al., 2011; Salimpoor et al., 2011, 2013; Zuckerman et al., 2012; Istok et al., 2013; Bogert et al., 2016). Indeed, even though “rock musicians never ask if a composition is aesthetically valuable,” they are still keen in evaluation “if it *sounds good*,” as observed by Račić (1981, p. 200, emphasis from the original). The study of aesthetics would be too narrowly construed if questions of what “sounds good” were ignored.

The same point can be made in the case of visual aesthetics. As pointed out by Redies (2015), the creation of visual beauty is not limited to any particular style, method, genre, or form, such as color, shape, luminance, texture, edges, or depth cues. A wide variety of materials can be used to create visually appealing objects. This suggests that the neural processes associated with aesthetic experience are not restricted to any particular feature (or corresponding neuronal circuits) or to a particular genre or style. We propose that the same might be true of music.

## Global aesthetic sensory qualities

We argue that global computational properties play a role in music aesthetics, and provide an overview of what we consider some of the most relevant global sensory properties to be. We also discuss previous research in the aesthetic of music that highlights the importance of such features. This review will be limited to global sensory properties, thus for the sake of clarity we ignore properties relating to culture, history or listeners' cognitive biases that are also supposed to play a role in a musical aesthetic experience (Chapman and Williams, 1976; McPherson and Schubert, 2004; Brattico, 2009–2010). The next section is dedicated to the discussion of the possible role of global properties in brain processing. As a provisional entry to this topic, note again that it is well-known that both musicians and non-musicians do in fact use global and “holistic” notions, such as “beautiful,” “melodious,” “rhythmic,” “touching,” “harmonic,” “peaceful,” “atmospheric,” “calming,” or “versatile” when describing the personal aesthetic value of music (Jacobsen, 2004; Istok et al., 2009). Most if not all of these concepts describe abstract impressionistic and holistic properties characterizing the piece as a whole, and are not strictly dependent on (although they might interact with) music-theory based local notions, such as intervals or chords. The same point can be further appreciated by noting that aesthetic perception is in no way tied to the Western music genres, but applies equally well to non-Western music. Indeed, when we look art and aesthetics as a whole, it is true that “some kind of aesthetic activity is apparently a feature of all the 3,000 or so distinguishable cultures that are to be found on the earth's surface,” as observed by Berlyne (1971 p. 27). Hence, we believe that aesthetics or aesthetic theories should not be tied with any particular style, genre, or music-theoretical notion.

The key distinction between *global* and *local* features is best elucidated by first looking how they are used in the study of visual aesthetics, and then by extending the notion to the domain of music and auditory aesthetics. In the study of vision and visual beauty, local properties of an image constitute the individual parts of the image, such as local color patches, lines, shapes, contrast, textures, surfaces, or other visual elements. Such local elements can be either *formal*, consisting of various non-conceptual or non-representational forms, or *content-based*, consisting of elements that represent something else. Examples of the former elements are color patches, lines, and textures, of the latter faces and objects. Early processing of visual information is predominantly local, as each local point in an image is projected tonotopically to a point in a visual representation (Wurtz and Kandel, 2000). As the information processing continues, however, the local features are integrated into a whole percept, or Gestalt, that “puts each pictorial element in perceptual relation to the other elements in the artwork” (Redies, 2015, p. 6) and thus integrates the various local elements together. It is that whole Gestalt that, according to many vision researchers, is relevant to the appreciation of beauty (Ramachandran and Hirstein, 1999; Zeki, 1999). Thus, the “Global structure refers to statistical regularities in large parts of the image or in the entire image, for example the spatial frequency content of the image, the kurtosis of its luminance values, overall complexity of self-similarity” (Redies, 2015, p. 4). Hence, it is not generally possible to take a piece of art, break it into pieces and then reassemble it back in random order while automatically preserving its artistic qualities. Formulated in this way, the distinction between global and local properties becomes relative. A painting on a wall constitutes a local feature of an even more global space, the whole wall. A modern artwork may consist of a red spot on a white background, making what in some other context would constitute a local feature a global one. These problems are kept under control by minimizing the impact of the context, for example, by framing and isolating the artwork in various ways from its natural surroundings and other objects of interest.

The global-local distinction elucidated above applies to music. In music, the local features can be best illustrated by the musical score, by separate tracks in a digital audio workstation (DAW), or by separating the performance of each band member from the rest, where each note/tone or interval appears in isolation and is mapped to the production of physical sound with certain timbre- and rhythmic characteristics during performance and/or recording. A note carries local information concerning timbre (instrument), dynamics (loudness), pitch, pitch changes (vibrato), duration and internal change (staccato, marcato, legato). The notes are further integrated into melodies and harmonic structures and relations that can still constitute local features. In a typical multi-instrument composition, several melodic themes are weaved together to create a sense of harmonic and melodic development. A local feature can be detached from the whole musical piece simply by muting it, or by muting a whole track in a sequencer. For instance, a melody can be changed, even dramatically, by changing the pitch or duration of just one note, and this produces fast reactions in the brain (such as the mismatch negativity, MMN, and the P3a responses) reflecting both an automatic processing of the change and the reorienting of involuntary attention toward the unexpected event. In turn, we propose that global musical features involve the composition as a whole, being synthetized from individual local features as they get summed into an integrated Gestalt. One can refer to the totality of all local features as the overall “musical texture.” Although it is possible to attend to each local part selectively, this is arguably not the norm and restricted to certain artificial contexts. The idea of removing, let alone freely reassembling, some parts from a composition is quite alien to the normal production and consumption of music. Thus, as in the case of visual art, we believe that it is the totality of all such elements that determine their artistic and aesthetic value. For instance, in the production of commercial-grade music global auditory features are manipulated during the final mastering process by using limiters, compressors, equalizers, and other dynamic and spectral processors. In the same vein, listening to any of the tracks or sounds in a musical piece in isolation will typically not lead to a positive, impressive aesthetic experience; it is their combined sum that will do that. Below we provide examples of aesthetically relevant music-specific global features.

### Distribution of spectral energy

An important aesthetic quality of music concerns the distribution and dynamics of its spectral energy. An aesthetically appealing sonic object is typically created by controlling the balance of its spectrum energy along several important dimensions such as (i) frequency, (ii) space, and (iii) time, as discussed below. “The goal” in sound engineering and mixing is “to get every aspect of the track to balance: every pitch and every noise; every transient and every sustain; every moment in time and every region of the frequency spectrum” (Senior, 2011, loc. 4904). Orchestral and other groups of instrumentalists adhere to the same principle, explicitly, or implicitly. It is crucial that, even in a loud performance typical of rock music, for example, the instruments are balanced.

In the *frequency* domain, we propose that the crucial balance is achieved by ensuring that the musical information is distributed throughout the whole audible frequency spectrum, and that the signal-to-noise ratio for each meaningful package of musical information (i.e., instrument, singer, instrument group or, more generally, a perceived sound source) is good enough so that no lower or higher level auditory masking intervenes. Indeed, the idea that efficient coding plays a role in human perception is supported by empirical evidence. Listeners must be able to hear all instruments in a distinctive way (not as a fuzzy auditory mess) even if they focus attention only on one of them, and thus these instruments have to live inside their own “safe space” in the spatiotemporal spectrum to avoid frequency masking, even when the music is composed out of digital samples of instrument sounds. They must furthermore appear controlled and consistent. Unaesthetic dynamical changes, conflicts and overlaps are routinely cleaned up by using filters, equalizations, compressors, and other techniques. In addition, often pop/rock and jazz music thrives to fill in the whole frequency spectrum by having “bottom end” (bass, kick drum), “high end” (hihats, cymbals, high pitch sounds), and “middle range” (singers, guitars, snare drums) instruments playing simultaneously (Corozine, 2002). Systematic empirical evidence is scarce, but composers are aware that the complete lack of any of the here described components will lead into a distinctive impairment in the aesthetic quality of the overall sound.

The unaesthetic masking phenomenon referred to above might result from the biological architecture of the human auditory system. The auditory system works by decomposing the signal by several narrow cochlear filters, or critical bands, each spanning a relatively small frequency range. The number and constitution of these bands is derived from psychoacoustic masking experiments, so that they capture the upper bound on the human frequency discrimination ability (Zwicker, 1961; Moore, 2012: Ch. 3). For the most part the frequency range increases logarithmically as a function of the central frequency, and the amplitudes of the resulting filters undergo nonlinear basilar membrane compression such that they are less sensitive to higher amplitudes. Furthermore, the human ear is most sensitive to the middle frequencies around 1,500 Hz, while the sensitivity decreases for sounds with both lower and higher frequencies. The temporal resolution of the auditory system, however, surpasses that of the other senses. Indeed, temporal resolution is required in the processing of fast transients and other sound changes that occur in, e.g., natural speech (Plomp, 1964; Zatorre et al., 2002). Further processing takes place once the signal travels to the auditory cortex via several subcortical regions (Barbour and Wang, 2003). The implication is that there are limitations on how much frequency/temporal space each musical signal can occupy to be perceived distinctly and clearly by the human brain in relation to other, surrounding musical information. This is especially relevant in the context of complex auditory signals, such as speech or music. Professional audio engineers', music producers' and composers' aim for distinctiveness in the sound can be interpreted as suggesting that avoidance of low- and high-level auditory masking contributes to sonic aesthetic experience. The notion is global, however, because it concerns the musical piece as a whole: how distinct various instruments and musical signals are perceived in relation to each other.

In the *space domain*, several techniques such as panning, reverbs, filtering, delays, filtering, and pre-delays are manipulated to position the musical information distinctively within the spatial field. This positioning is achieved by modeling the way the human brain encodes spatial information from the acoustic signal (Zahorik, 2002). For example, when a musical instrument is embedded within a space by using an artificial or natural reverberation, a few milliseconds of pre-delay in the reverberation can change the perceived distance of the source: a reverb with no pre-delay will position the source to the back wall of the virtual space, while 20–30 ms pre-delay will bring it closer to the listener. This models the time the reflected (reverberated) sounds will normally lag behind the direct sound. Similar manipulations are used in experiments testing the neural abilities for discriminating sound sources. Notably, these abilities, relying on the fast elaboration of differences in the incoming signal as compared with the environment at the level of the auditory cortex are very sensitive to even small variations of spatial location (Colin et al., 2002; Roeber et al., 2003; Altmann et al., 2014). But the spatial interpretation of music is global in the sense that it concerns the relative position of the listener to that of the sound source and the environment, whether these are real or virtual. The spatial dimension is also used when positioning sound sources to different locations within a virtual space in order to keep the said sources sufficiently distinct from each other.

In the *temporal domain*, the dynamical qualities of individual instruments (e.g., transients) and the whole song structure are controlled to create a sense of music development and to adjust for the inevitable sensory habituation. “In a lot of cases in commercial music,” Senior (2011) observed, “you want to have enough repetition in the arrangement that the music is easily comprehensible to the general public. But you also want to continually demand renewed attention by varying the arrangement slightly in each section” (loc. 2523). For example, to maintain listeners' attention one is advised to “provide some new musical or arrangement diversion every 3–5 s to keep listener riveted to the radio” (loc. 2592). Thus, the balance between repetition/regularity and novelty, much discussed in the study of aesthetics and supposedly following an inverted U-shape function (Berlyne, 1971), does not concern only rhythm (Vuust and Witek, 2014; Witek et al., 2014) or melody (Green et al., 2012), but is related to a change of any kind, including changes in the global musical texture.

### Musical texture

The term “musical texture” refers to the way that local musical features, such as rhythm, melody, and harmony are integrated in a whole composition and, ultimately, into a whole Gestalt percept in the listeners' brain (e.g., Meyer, 1956 p. 185–196). Texture is an elementary consideration in both arrangement and orchestration, processes that aim for crafting an aesthetic output from several local themes such as rhythm, melodies, counter-melodies, and harmony. The same four-way voicing, such as an arrangement for four saxophones, may have quite different textures if it is arranged in parallel compared to when the individual voices are allowed to cross one another. Music that strongly relies on music theory properties can benefit immensely from properties of the texture, as in the case of popular or film music, suggesting that texture alone can be a crucial component in determining an aesthetic response to music.

While the study of texture perception is a lively topic in the domain of vision, with by now a long tradition (e.g., Julesz, 1962), very little comparable research exists in the case of auditory modality. In one study, McDermott and Simoncelli (2011) constructed a physiologically realistic model of the auditory system, which they provided with samples of various repeating sound textures, such as rainstorms, insect swarms, river, and wind, and then used the model to extract biologically plausible time-averaged statistical properties from the textures. These statistical measures represent high-level descriptions of the sound source. They were used to synthetize the same texture sounds from white noise, and the results were compared against the natural sounds in an experiment by using human participants. Sound synthesis was either biologically realistic or unrealistic. The logic of the experiment was to use human performance as a way to benchmark the biological plausibility of the model. For example, when the synthetic sounds were indistinguishable from the natural samples by the human participants, it could be assumed that the generative model closely matched that of the human auditory system. When the participants noted marked differences with the original texture and the synthetic one, we can reason that the model did not mimic the human auditory system. A clear contrast emerged between realistic and unrealistic assumptions, suggesting that the human auditory system might indeed extract statistical properties of the sounds to encode and represent its global textural properties. For further experimental evidence that human auditory system utilizes time-averaged statistical processing to represent textures and other global features of sounds, see McDermott et al. (2013). In the latter study, the authors proposed a functional explanation for their findings, suggesting that statistical averaging is used by the auditory system to overcome memory limitations. The evidence that the auditory system uses statistical time-averages is encouraging for our hypothesis that part of the music aesthetic experience relies on global sensory properties, because it provides empirical justification for the claim that such global features could play a direct role also in auditory perception. These studies go further by proposing that there are neuronal populations within the auditory pathway that are specifically dedicated and tuned to detect global statistical properties of the auditory signal. This raises the possibility that the immediate aesthetic value in certain global sensory properties would be directly assessed by low-level modules in the brain, rather than being assembled only later when the isolated local features are merged into a whole percept. Whatever the case, we encourage studies for testing the hypothesis that, as in the case of visual textures, musical texture would play a comparable role in the aesthetic perception of music.

### Expressivity

Another relevant global quality that affects the aesthetic appeal of a sonic object is its music-emotional impact or expressivity (Robinson, 1994; Gabrielsson and Juslin, 1996). While playing synthetized chord sequences or sinewave tones in isolation and in temporally exacting sequences can indeed evoke emotions and aesthetic judgments due to their ability to represent elementary harmony relations, there is a difference between fully mechanized, synthetic version and humanly played orchestral version of the same piece such that the latter will be perceived as more aesthetic than the former (Seashore, 1929). The “humanness” in the performance of a real human being is especially relevant to the perceived emotional character of the performance. This indicates that there are global sensory features that exhibit a direct causal relationship with human emotions and the “emotional centers” of the brain (Koelsch, 2014). What these features are remains elusive, but the study of visceral affective reactions to music, such as chills, has revealed that there indeed exist prototypical sonic qualities that tend to evoke strong emotional responses in listeners. Laeng et al. (2016), for example, mentions properties such as the beginning of a piece, an entry of an instrument or human voice, melodic appoggiaturas (“extra notes or ornaments”), dynamic changes in loudness, surprising harmonic changes, and sustained high-pitch tones of instruments or voice, among other techniques (see Sloboda, 1991; Panksepp, 1995; Gabrielsson and Juslin, 1996; Rickard, 2004; Grewe et al., 2007; Gabrielsson, 2011; Branković, 2013). If we compare raw mechanical and synthetized instrumentation to that of a real human performance, a complex of dynamic and timbral differences emerge such that latter contains a continuous stream of changes in dynamics (attack, sustain, release), pitch (vibrato, true legato), timbre and spectrum, pauses (breathing, bowing), and many others.

### Tempo and mode

Researchers have shown that global properties such as tempo and mode (minor or major) influence preference and liking, possibly due to their association with basic emotions such as sadness and happiness (Hevner, 1935; Dalla Bella et al., 2001; Pallesen et al., 2003; Khalfa et al., 2005; Hunter et al., 2008; Schellenberg et al., 2008; Nieminen et al., 2012). Slow tempo and/or minor mode are associated with sadness, while fast tempo and/or major mode with happiness, the latter receiving more positive liking ratings (Husain et al., 2002). Tempo, meter and mode are global properties in the sense that they describe, not individual instruments or parts, but large segments of the compositions, or indeed the composition as a whole. Mode, for example, characterizes the underlying key (minor vs. major) upon which the composition, or a segment of the composition, is based on. It also describes the tonal center of the piece that the listener will expect the musical development to return periodically through tension and relaxation. When the mode is in major, the music sounds happier overall than when it is in the minor key. Similarly, the meter of a song, be it, e.g., a waltz (3/4) or a march (2/4) fundamentally influences the mood of the song.

### Other properties and experimental expectations

In addition to the examples above, there are other global properties that are known to affect the rewarding responses to music, such as exposure or familiarity (Heingartner and Hall, 1974; Bornstein, 1989; Peretz et al., 1998; Pereira et al., 2011) and groove (Janata et al., 2012; Sioros et al., 2014; Vuust and Witek, 2014; Vuust et al., 2014; Kilchenmann and Senn, 2015; Fitch, 2016). Exposure and familiarity, in particular, affect liking in an inverted U-shape function, so that repetition will first lead to increased preference but the effect disappears if too much repetition is administered (Green et al., 2012).

In sum, alongside the more local and analytical musical features, there are several types of global sensory properties that seem to play a role in the creation of an aesthetic experience of music. One group of properties involves the distribution and dynamics of spectral energy. In aesthetically appealing music, each instrument or meaningful musical signal should occupy its own spectral space in terms of its frequency-based, spatial and dynamical dimensions in order to control auditory masking. A closely related aspect of musical aesthetics is constituted by musical texture, which refers to the overall sound that results from the combination of its local parts. Arrangement and orchestration are two ways musical texture is created, with much of the consideration having to do with distinctiveness and hence ultimately spectral dynamics. We also discussed expressivity, mode, tempo, familiarity and groove, all linked to emotions, as other possible examples of aesthetically relevant global properties.

All the global sensory properties discussed above apply equally well to Western and non-Western music and musical styles. Thus, a spectrally and spatially rich musical texture can be generated by manipulating digital instruments in a modern studio, for instance, in Western style for a pop music project, as well as by producing Balinese gamelan music in its natural surroundings. This is reasonable, since aesthetic responses are not a privilege of Western music and thus should not be explained as outcomes of only one musical genre or style.

The hypothesis linking global properties to aesthetic responses renders itself naturally to empirical experimentation. For example, the balance in spectral energy distribution can be rigorously manipulated at the stimulus level. This can be achieved by removing and/or adding sonic components at specific locations within the spectrum, irrespective of their representational or other content. If our hypothesis is correct, then such manipulation should lead into prominent changes in, e.g., aesthetic pleasure of such objects irrespective of their higher-level content (i.e., comparison between Western and non-Western music). Another relevant consideration comes from the recent naturalistic paradigm, discussed in detail in the next section, that is suited for addressing global properties particularly well. We return to the experimental issues in the section Sensory Aesthetics as Immersion and Arousal, where we discuss the present hypothesis from a neuroaesthetics viewpoint.

## The naturalistic paradigm for studying global sensory properties

Some recent work toward analyzing musical stimuli in terms of their global sensory properties have been done thanks to the introduction of the naturalistic paradigm in music research. In this paradigm, the participants are required to listen attentively to a whole piece of music while their brain signal is measured. Afterwards, their brain signal is analyzed as a time-series in combination with qualities obtained by exploiting knowledge from music information retrieval (MIR), namely acoustic parameters that are relevant for identifying musical genres and extracting timbral, tonal, and rhythmic information from musical pieces. Specifically, the brain signal measured with functional magnetic resonance imaging (fMRI; Alluri et al., 2012, 2013; Burunat et al., 2016) and with electroencephalography (EEG; Poikonen et al., 2016a) has been analyzed by extracting acoustic variables from the music by using the MIR Toolbox (Lartillot and Toiviainen, 2007). This approach is based on the assumption that global computational sensory properties in naturalistic musical stimuli provide a useful window not only to technological applications but also into our appreciation of music and its neural implementation. Most of the relevant properties in these studies are spectral in nature and concern the way in which auditory energy is distributed both in frequency- and time-domains (see Table 1). This approach itself is a derivative of a larger research agenda of MIR that is aimed at extracting musically relevant information from whole musical pieces by using computational and statistical techniques (see Peeters, 2004; Moffat et al., 2015). MIR algorithms extract global features from the audio signal that are furthermore distantly related to the global features we claim could be relevant to aesthetics.

**Table 1:** **Acoustic features used in Alluri et al. (2012)**.

**Feature**	**Description**
Loudness	Root mean square energy: the square root of sum of the squares of the amplitude.
Zero crossing rate	Number of time-domain zero crossings of the signal per time unit.
Spectral centroid	Geometric center on the frequency scale of the amplitude spectrum.
High-energy—low-energy ratio	Ratio of energy content below and above 1,500 Hz.
Spectral entropy	The relative Shannon entropy, which measures peaks in the auditory spectrum.
Spectral roll-off	Frequency below which contains 85% of the total energy.
Spectral flux	Measure of the temporal changes in the spectrum.
Spectral spread	Standard deviation of the spectrum.
Spectral flatness	Wiener entropy of the spectrum, which measures as the ratio of its geometrical mean to its arithmetical mean.
Sub-Band flux	Measures the fluctuation of frequency content in 10 octave-scaled sub-bands.
Roughness	Estimates the sensory dissonance.
Mode	Strength of major or minor mode.
Key clarity	Measures tonal clarity.
Fluctuation centroid	Estimates the average frequency of rhythmic periodicities.
Fluctuation entropy	Measures the rhythmic complexity.
Pulse clarity	An estimate of the clarity of the pulse.

*Of these, six clusters (Fullness, Brightness, Timbral complexity, Key clarity, Pulse clarity, Activity, and Dissonance) were created for the study by using principal component analysis (PCA). Detailed description of the features can be found from the original source and from the MIR Toolbox manual*.

Particularly, in Alluri et al. (2012) the authors asked the participants to consciously listen to a musical piece (*Adios Nonino* by Astor Piazzolla) while their brain activation was simultaneously observed by fMRI scanning. The brain scans were correlated with statistical properties extracted from the song, such as overall loudness, spectral centroid, high-energy-low energy ratio, spectral entropy, spectral flux, and tonal clarity (see Table 1). Once these features were extracted from the whole song, the original 25 features were reduced into 9 by performing a principal component analysis (PCA) on the resulting song-wide feature vector. The remaining cluster features were global features such as Fullness, Brightness, Timbral complexity, Rhythmic complexity, Key clarity, Pulse clarity, Event synchronicity, Activity, and Dissonance, of which two (Rhythmic complexity and Event Synchronicity) were removed as they did not correlate with participants' subjective assessment in a separate behavioral experiment. Of the remaining six global sensory properties, the authors showed that their presence and absence in the musical stimuli indeed did correlate with brain activity. For example, the timbral features (Fullness, Brightness, Timbral complexity, and Activity) were associated positively with activity in the superior temporal gyrus (BA 22) bilaterally and the cerebellum, and negatively with several regions, such as the postcentral gyrus (BA 2, 3), the left precuneus (BA 7), and the inferior parietal gyrus (BA 40). The study shows that such global statistical features do play a role in the musical experience and are indeed meaningful from the point of view of processing of music in our brains.

It remains to be seen, however, whether this approach can be applied to the study of aesthetics. Although, the statistical properties used in our previous studies (Alluri et al., 2012, 2013; Burunat et al., 2016) may be too coarse to be directly relevant for aesthetics, in particular when it comes to the masking problems, the approach is consistent with the hypothesis advanced here. Moreover, the hypothesis that any of such properties were relevant to aesthetics can be tested empirically by correlating the presence of such properties to that of listeners' subjective liking. Promising initial attempts toward that direction, namely combining the fMRI timeseries with continuous or discrete ratings have been made by Trost et al. (2015) and Alluri et al. (2015).

Several challenges must also be met when applying this naturalistic paradigm. Although it allows researchers to use realistic music stimuli, the listening conditions are less than optimal, especially in a fMRI setting, in which noise saturation, low temporal resolution and the risk of false positives in the results (Eklund et al., 2016; Liu et al., 2017) pose considerable methodological challenges to our approach. Even if replicability of brain responses to musical features using the naturalistic paradigm has been shown (mainly for timbral features; Burunat et al., 2016), the concerns for applying the current approach to fMRI data might present bottlenecks that are hard to circumvent. A promising direction would be to utilize silent neurophysiological methodologies with millisecond temporal resolution, such as magnetoencephalograhy (MEG) and/or electroencephalography (EEG). Two papers have obtained neural correlates of MIR features using EEG signals (Poikonen et al., 2016a,b) and we are studying the application of this approach to MEG data, which allows also a spatial resolution that is almost comparable to that of fMRI. Moreover, we do not wish to imply that this methodology be restricted to brain-imaging settings. It may be applied to behavioral experiments, and indeed many studies done in the naturalistic paradigm do involve behavioral components. In such experiments, participants are asked to evaluate naturalistic stimuli continuously, for example, by providing on-line rating or feedback of the music they are listening (Coutinho and Dibben, 2012). Also, global sensory qualities of a naturalistic stimuli can be independently manipulated in behavioral experiments in order to examine the aesthetic effects of such variables. In our view, it is possible that the optimal results are obtained by utilizing a combination of behavioral and brain-imaging methods. In such hybrid paradigms, many methodological restrictions of purely brain-imaging paradigms can be circumvented by applying behavioral methods, while the brain-imaging studies can provide detailed anatomical, physiological and time-sensitive data unavailable by using behavioral methods alone.

## Sensory aesthetics as immersion and arousal

In this section, we consider several possible neural explanations for the link between global sensory qualities and aesthetics. This approach is motivated by the fact that, if global sensory properties indeed are pertinent for the creation of an aesthetic experience, then there must be something in our brains, “some fundamental characteristics of the human nervous system” (Berlyne, 1971 p. 29), that explains that fact. What these fundamental characteristics are indeed constitutes a perennial problem of neuroaesthetics. The notion of global sensory properties could provide a contribution to this debate.

Historically, the search for a common aesthetic quality goes back at least to Bell's work on the aesthetics of art (Bell, 1914). Bell proposes that all art, and especially visual art, shares a universal time- and culture-independent “significant form” that is associated with aesthetic emotions. Bell thought that the form arises from aesthetic laws pertaining to the configuration of visual features such as lines, shapes, and colors. For Bell, a crucial test for separating aesthetic art from other stimuli was its universality and time-independence: a genuine aesthetic art should be independent of time, culture, and era.

Zeki (2013) provides a modern interpretation of Bell's theory. He begins from the well-known organizational properties of our visual system, according to which the neuronal processing of visual stimuli is distributed over several quasi-independent modules in the brain, each processing its own specialized domain (movement, colors, lines, faces, direction, and such), and then proposes that each of these modules “have a certain, primitive, biologically derived combination […] of elements for the attribute that it is specialized in processing, and that the aesthetic perception […] is aroused when, in a composite picture, each of the specialized areas is activated preferentially” (p. 10). Aesthetic perception, according to this hypothesis, has its origin in a “preferential” activation pattern of the early sensory areas specialized in visual perception that will then lead into the activation of interest- and motivation-related brain areas and hence also to an experience of emotion, beauty, and preference (Sachs et al., 2016). This provides a neurobiological interpretation of Bell's original idea. The “significant form” would refer to the fact that some type of preferred activity occurs in various visual regions of the brain, as if each such module would have its own aesthetic principles. Artists are professionals who “create forms that activate the relevant visual areas either optimally or specifically [and] in a way that is different from that obtained by stimuli that lack the significant configuration” (Sachs et al., 2016 p. 9).

We see certain similarities to the case of music. Playing back a stereo track in mono takes away some aspect of its appeal, much in the same way as removing all reverb from a recording makes it dull and lifeless. We hypothesize that this may be because the neural systems wired to detect direction and distance of sound sources are not activated in a natural way, or they are not activated at all. If the spectral energy is further reduced by, say, removing musical material from frequencies below some threshold 500 Hz, the music becomes thinner and, again, loses part of its appeal. The neuronal systems registering lower frequencies receive no input, and therefore contribute nothing to the overall percept. The idea of avoiding too much repetition by introducing constant change derives from the same source: a dull, repeating music ceases to command our attention. In addition, if a musical piece performed by real human beings is replaced by machines mechanically playing sinewave instruments, then the performance loses some of its emotional connotations and, again, some neuronal processes linking auditory signals with emotions that would otherwise be engaged are not involved. Thus, as observed by Baugh (1993), rock music “aims at arousing and expressing feeling” (p. 23) in the listener, which we believe holds the key to sensory aesthetic experience. Music, like vision, is a composite of several qualities (direction, distance, depth, emotion) processed by semi-independent modules in our nervous system, while each such module responds to its own signature properties in the stimuli. It might be that, as in the case of vision, aesthetic appeal originates in a concerted and balanced activation of all these modules. The global aesthetic properties in music, specifically, are aimed at optimizing the presence and balance of these qualities to keep different neural structures in the brain in a “preferential” activation and connectivity state. This brain state would, according to our hypothesis, lead to “immersion” or arousal in the listener, resulting in a rich, holistic experience (Brattico et al., 2013).

One way to refine this idea is to build on Berlyne's (1971) seminal work on aesthetics and arousal. The notion that aesthetic experience can be traced back to immersion, and especially arousal, was the cornerstone of Berlyne's work on the psychology and biology of aesthetics (e.g., Berlyne, 1971), who in turn followed much of the spirit of Fechner's (1876) pioneering work. Berlyne's main proposition was that the aesthetic experience, and aesthetic pleasure, derives from a change in organism's arousal level. The change could involve decrease (relaxing, tension reduction) or increase (excitement, expectation) of arousal level, and both could be triggered by several properties, among them novelty, surprise, complexity ambiguity for heightened arousal, and repetition, familiarity for reduced arousal levels. The global sensory qualities point toward the same direction. Thus, a sonic object evoking spatial and affective cognition, commanding the whole energy spectrum, and holding listeners' attention will lead to an immersive experience and continuous arousal: by introducing small changes and crafting a careful “building up” the artist creates a musical piece that avoids sensory habituation that would otherwise reduce its impact.

The Fechner–Berlyne approach has been subject to criticism. Their work belonged to the behaviorist-reductionist framework that sought to explain behavior in terms of bare stimulus-response principles. From such reductionist perspective, internal motivation, pleasure, or curiosity present themselves as near-paradoxical problems. An aesthetic object, in particular, is one that the organism is actively seeking to experience, and thus it presents a particularly difficult problem to explain. Berlyne's theory was an attempt to answer this problem. Many of his most strong critics, however, came from a different, humanistic-philosophical tradition involved with history, art criticism and philosophy, in which behaviorist problems played no meaningful role, and from which the whole enterprise appears unnaturally narrow (see Margolis, 1980, for an example). Today such criticism plays a much less significant role (Zeki, 2014; Bundgaard, 2015). The question of what motivates people, and makes objects desirable for them apart from their possible ecological functionality, is as relevant today as it was then. Further, the idea of explaining human behavior in terms of stimuli, brain physiology, and motoric responses cannot be substituted wholly by speculative philosophy, cultural relativism, or art history; modern neuroscience has a role in explaining human behavior. Indeed, there exists a small but active research program inside the neurosciences that can be characterized as “neuroscience of aesthetics” (for recent reviews see Jacobsen, 2006; Chatterjee, 2011; Brattico and Pearce, 2013; Orgs et al., 2013; Chatterjee and Vartanian, 2014, 2016; Pearce et al., 2016). Berlyne was in fact well-aware of the neuroscientific advances of his day, and documents such matters extensively in his work. At the same token, it is also clear that no neuroscientific or naturalistic exploration can answer questions such as what, ultimately, is art, and what makes a piece of material constellation a genuine work of art instead of a, say, tool or random junk. This is because art is constituted by several non-appearance properties such as its history, intention, sincerity, and normativity, and not everything beautiful or appealing can be said to be art (Bundgaard, 2015). A naturalistic approach to aesthetics (Brown et al., 2011) will, therefore, pay a price in necessarily ignoring many aspects of art that we would regard as important in other contexts.

Berlyne (1971) proposed that the main categories of stimuli that can modulate arousal, and hence in his theory also involve aesthetic appreciation, fall into three distinction categories: psychophysical, ecological, and what he called “collative” (also “structural”). Psychophysical qualities refer to low-level sensory features and changes in such qualities. He mentions in this connection the fact that more intense stimuli are normally interpreted as more arousing. Ecological variables refer to stimuli that are directly associated, either innately or by means of learned association, with survival, pain, and pleasure. Finally, by the term collative or structural properties he means second-order properties that are arrived at by “summing up characteristics of several elements” (p. 69) that may be present simultaneously or could also be temporally distinct. Properties such as novelty, complexity ambiguity and surprisingness belong to this category. The global sensory properties discussed in the present work would, under this scheme, consist of a mixture of structural and psychophysiological properties: they are structural and global, in that they result from the summation of few or many individual qualities, but also sensory, in that they depend on the sensorium and are not constitutively affective or cognitive.

The immersion hypothesis, according to which aesthetic experience results from an activation of all or many brain regions specialized in the processing of the stimuli, leads to testable hypotheses, and empirical predictions. Vartanian and Goel (2004), for example, report that increased preference in the perception of visual art correlates with increased activation in the visual areas of the brain. If sensory immersion and arousal play a role in aesthetic perception, then the expected outcome is precisely that we should attest a positive correlation between aesthetic preference and the activation of the various brain areas involved in the processing of the stimuli. This prediction was also confirmed in a meta-analysis, likewise reporting an association between visual aesthetic experience and a wide-spread rather than localized brain activation (Boccia et al., 2016). In the case of music, the prediction is that the removal of relevant features, whether spatial, emotional, or spectral, should lead into a marked decrease both in the brain activation and in the aesthetic judgment. Crucially, our hypothesis predicts that this effect should not depend on local features, and should be observed entirely irrespective of musical genre, style, or (representational) content. If, in other words, the aesthetic balance in sensory qualities is achieved by means of immersion, itself based on the concurrent activation of the relevant brain regions, then what matters is the activation itself and not the particular local features present in the activating stimulus. This hypothesis could thus further be tested by invoking experimental top-down effects that suffice to satisfy the activation condition without the presence of concrete stimulus.

However, this hypothesis predicts, if interpreted in a too simple way, that increasing the amplitude of any or all such features should always lead to increased liking. Oversaturated objects, such as overly loud music or pictures with bright colors, are not perceived as beautiful; instead, they can be perceived even as painful. Too much reverb, stereo widening or emotional expressivity makes the music incomprehensible and “wishy-washy.” This question has always puzzled those trying to understand aesthetic perception. Berlyne's solution was to assume that stimulus levels beyond a certain moderate cutoff point would begin to active “aversion systems” that are associated with a negative outcome (danger, unpleasantness). Zeki (2013) discusses this problem and points out that the determining factor cannot be the strength of the activation as such but, rather, there must be some quality in the original signal that prompts the positive response. He provides another interpretation of these results, according to which “it is not the strongest or maximal activity that correlates with preference but rather a specific activity that becomes optimal when stimuli of the right [aesthetic properties] are viewed” (p. 10). Hence, we are back at Bell's mystery: there is an unknown quality in the stimulus that is preferred by the various regions in the brain specialized in processing that type of stimuli.

The case of music provides another possible interpretation. It is an established fact that the masking effect on one sound over another is amplified by the amplitude of the former. Thus, as the sound is increased in amplitude, the range of frequencies it will mask will also increase. Moreover, if we are presented with a piece of music in which one instrument is associated with overwhelming volume, our brains will attempt to adapt to the situation by attenuating the overall level. This will further reduce the perceived relative amplitudes of the rest of the musical information. Finally, the problem might not be as severe if the amplitude of all sound sources is increased in tandem, which corresponds to an increase in overall volume. Thus, we might be dealing, not with overall amplitude, but with relative amplitudes. It is possible that the reason why balanced performances instead of overly saturated ones are crucial for auditory aesthetics is because the former specifically avoids unaesthetic masking and thus keeps the musical sources distinct. This hypothesis could be tested experimentally. If the problem with amplitude concerns relative amplitudes and/or masking, then the same negative effect on aesthetic experience could be achieved by using other types of masking (noise masking) and/or also by decreasing an amplitude of a sound source relative to other sound sources.

There is another intriguing possibility. The causal relation between global sensory properties and aesthetics could be further captured in terms of the processing fluency hypothesis, as proposed in the domain of visual aesthetics (Reber et al., 2004; Babel and McGuire, 2015; Forster et al., 2015). For instance, if the crucial feature concerns the distinctiveness of each musical source in the absence of feature masking, then it is possible that the phenomenon reduces further to the notion of processing fluency (Reber et al., 2004), namely the relation between a positive aesthetic response and the ease of processing in encoding and representing e.g., distinct sound sources. This hypothesis predicts that aiding the encoding of music and its sound sources by visual means, for example, by exhibiting the performance itself, should increase the aesthetic appeal of the piece irrespectively of whether the sound sources are overlapping or not. On the other hand, a dull, unsaturated but fluently processed sonic object might be less appealing than a complex one that incorporates the whole frequency and spatial spectrum, an obvious problem for the fluency hypothesis. This problem could be solved by combining the immersion hypothesis with the processing fluency hypothesis. Accordingly, perhaps an aesthetic appreciation requires a concerted and concurrent activation of all the relevant modules that participate in the processing of the stimulus, as assumed by the immersion hypothesis, but with the additional requirement that each module has to be able to process its input in a fluent and efficient manner, as assumed by the processing fluency theory. Under this hypothesis, the masking phenomenon linked with musical aesthetics would be interpreted as a distracting event that hinders fluent processing in any of the relevant submodules.

If we, instead, assume Zeki's hypothesis that there are “significant forms” that, by causing the various sensory submodules to enter their “preferred states,” lead into aesthetic appreciation, then a rigorous definition of “significant form” is required. Zeki (2013) discusses the example of human faces in this connection. Humans have an inborn preference for perceiving, representing, and interpreting human faces, and there are specific neuronal resources dedicated to this task. These visual systems respond selectively to the properties of human faces and, moreover, some such features are perceived as more attractive than others. There are biological and evolutionary reasons why such preferences would inhabit our visual system, and the same phenomenon of “mate selection” is observed throughout the animal kingdom. The same argument can be found from several views concerning visual aesthetic, cited earlier in this paper. The idea is that the global sensory properties are shared with the biologically preferred visual images, such as natural landscapes or potential mates, which would then explain artistic preferences as a halo effect of the originally more mundane mechanism. While we do not wish to propose that all aesthetic perception derives from preferred tuning of the various sensory systems for mate selection, landscape detection, or healthy nutrition detection, this view provides a plausible argument for the *existence* of such mechanisms. Preference of certain types of mates, environments, foods, and tastes, for example, is something that our brains must be hardwired to do, although also learning and cultural exposure have an effect, while it is possible that such preferences spill over non-functionally to the perception of many types of objects, and even to abstract objects such as music. This hypothesis could be labeled as the *ecological hypothesis*. It has been pursued in the domain of vision by examining whether global statistical sensory properties of ecological stimuli, such as natural landscapes or faces, lead into aesthetic experience when they are embedded in the context of abstract art objects or other visual stimuli. These experiments could be replicated in the case of music by extracting global statistical sensory properties from ecological sounds (wind, rain, human voice, crying, laughing) and replicating then synthetically in music or in music-type stimuli to determine if their aesthetic value can be modulated.

## Conclusions

We put forward a research agenda for studying holistic qualities of musical objects that likely play an important role in creating an aesthetic response in the listener. We propose that these global features are statistically extracted from the stimuli by our auditory system—or, rather, by some subsystems (McDermott and Simoncelli, 2011; McDermott et al., 2013)—and then passed on to high-level processing, ultimately leading to the main outcomes of a musical experience, namely aesthetic judgment, emotion and conscious liking, or preference (Cela-Conde et al., 2011; Brattico et al., 2013). A shift of paradigm from conventional studies using artificial stimulation, block design, and subtraction analysis methods toward novel naturalistic paradigms with non-conventional analysis methods based on MIR combined with brain time series is called upon to accurately measure and determine the effects of global properties on brain functioning and behavior. We also discussed several possible neuronal implementations of this general hypothesis: the immersion hypothesis, processing fluency hypothesis, and the ecological hypothesis. The immersion hypothesis claims that aesthetic experience results in a concerted activation of many or all critical brain regions involved in the processing of the stimuli, irrespective of other stimulus content; the processing fluency requires that the stimuli can be processed effortlessly by the brain; and the ecological hypothesis contents that the modules have to enter into a “preferred” neural state that is further determined by ecological conditions. Another possibility is that they all play a role.

## Author contributions

PB and EB conceived the hypotheses of this paper. PB wrote most of the manuscript whereas EB wrote some parts of it. PV edited the manuscript and contributed to financing the work.

### Conflict of interest statement

The authors declare that the research was conducted in the absence of any commercial or financial relationships that could be construed as a potential conflict of interest.
